# A Rare Case of a Brain Abscess With a History of Penicillin Allergy

**DOI:** 10.7759/cureus.97637

**Published:** 2025-11-24

**Authors:** Ryuichi Hirano

**Affiliations:** 1 Pharmacy, Sapporo Azabu Neurosurgical Hospital, Sapporo, JPN

**Keywords:** brain abscess, fusobacterium necrophorum, metronidazole, odontogenic infection, penicillin allergy

## Abstract

The administration of β-lactam antibiotics is the standard treatment for brain abscesses. However, there are no detailed case reports of brain abscesses with a history of allergy to β-lactam antibiotics. Here, we present the case of a patient with a penicillin allergy who underwent successful treatment for a brain abscess without the recurrence of anaphylaxis symptoms. A 91-year-old man was admitted with a brain abscess. β-Lactam antibiotics were not considered to be suitable because the patient had previously developed anaphylaxis symptoms to penicillin antibiotics. Due to poor oral hygiene, anaerobic bacteria were suspected as the underlying cause of the brain abscess. Intravenous metronidazole (MNZ) therapy was initiated after intracerebral drainage. *Fusobacterium necrophorum* was isolated from a culture of pus obtained via drainage. We concluded that the abscess was caused solely by *F. necrophorum* because aerobic cultures of the abscess were completely negative, whereas *F. necrophorum* was isolated only under anaerobic conditions. Following 45 days of MNZ therapy, the patient was discharged without the recurrence of anaphylaxis. The learning point of this case is that physicians must confirm patient histories of antibiotic allergies and adjust treatment based on the information collected, particularly for patients with a history of penicillin allergy.

## Introduction

A brain abscess is a focal pyogenic infection of the brain caused by bacteria [1]. The incidence of brain abscess is approximately 0.4 per 100,000 people [2]. Despite recent advances in diagnostic technologies leading to favorable outcomes, the mortality rate for brain abscess remains high [3]. Therefore, physicians are required to perform neurological drainage and long-term antimicrobial therapy.

Adverse drug reactions are the most significant issue for patients requiring long-term antimicrobial therapy. Penicillin antibiotics are the most frequently reported causative agents of drug-induced hypersensitivity reactions [4]. Approximately 9.4% of patients administered penicillin antibiotics experience hypersensitivity reactions [5]. Most cases are mild skin reactions, such as rashes. However, patients with severe anaphylaxis require invasive interventions (e.g., intubation) by critical care physicians. Therefore, it is crucial to prevent anaphylaxis, particularly for patients admitted to facilities without intensive care physicians.

The standard treatment for a community-acquired brain abscess is the intravenous administration of a β-lactam antibiotic and metronidazole (MNZ) for six to eight weeks [6]. There are no detailed case reports of brain abscess with a history of penicillin allergy (PenA). Here, we describe the successful treatment of a brain abscess in a patient with PenA without the recurrence of anaphylaxis symptoms in a neurosurgical hospital that does not have intensive care physicians who manage anaphylaxis.

## Case presentation

A 91-year-old man visited our hospital’s emergency department (Sapporo Azabu Neurosurgical Hospital, Hokkaido, Sapporo, Japan) due to aphasia and dysarthria. On admission, his vital signs were as follows: blood pressure of 150/60 mmHg, SpO₂ of 95%, heart rate of 58 beats per minute, and body temperature of 36.4°C. His Glasgow Coma Scale score was 13. Table 1 shows the laboratory findings at the time of admission. MRI of the head revealed an abscess in the left frontal lobe. The patient was admitted for intracranial drainage. His underlying diseases were angina pectoris and periodontitis. The patient had previously experienced a rapid decline in blood pressure and loss of consciousness immediately after the intravenous administration of penicillin antibiotics. The previous physician diagnosed the patient with PenA based on the severity and immediacy of the symptoms that developed.

**Table 1:** Laboratory findings on admission.

Test	Result	Normal range
Creatinine	0.71 mg/dL	0.65–1.1 mg/dL
Creatinine clearance	48 mL/minute	90–120 mL/minute
Blood urea nitrogen	20.5 mg/dL	8–20 mg/dL
Aspartate transaminase	21 IU/L	10–40 IU/L
Alanine transaminase	12 IU/L	5–45 IU/L
Total bilirubin	0.6 mg/dL	0.3–1.2 mg/dL
Albumin	2.9 g/dL	3.8–5.2 g/dL
Sodium	139 mmol/L	135–145 mmol/L
Potassium	4.3 mmol/L	3.5–5.0 mmol/L
C-reactive protein	1.5 mg/L	Less than 0.3 mg/L
White blood cells	5,400/μL	3,500–9,700/μL
Hemoglobin	12.4 g/dL	13.6–18.3 g/dL

On the first day of admission, physicians consulted the infectious disease (ID)-specialized pharmacist regarding the use of antibiotics. As our hospital specializes in neurosurgery, there are no intensive care physicians who manage anaphylaxis. We selected the administration of non-β-lactam antibiotics to avoid the recurrence of anaphylaxis.

On the second day, intracranial drainage was performed. Clindamycin (CLDM) at 600 mg q12h was intravenously administered to prevent surgical site infection (SSI). Gram staining of the abscess was negative. No bacteria were isolated from the blood culture submitted on the same day. Following the procedure, we examined the underlying cause of the brain abscess. The patient had a tooth extracted due to periodontal disease two weeks before his admission. No prophylactic antibiotics were administered after tooth extraction. Due to poor oral hygiene and the history of tooth extraction, anaerobic bacteria in the oral cavity might have caused the brain abscess. Therefore, intravenous MNZ therapy at 500 mg q8h was initiated on day three because of its activity against anaerobic bacteria as well as its good penetration into the central nervous system. The ID pharmacist recommended that the physician administer vancomycin (VCM) in addition to MNZ based on the isolation of Streptococcus species in many brain abscess cases [1]. However, this recommendation was not accepted due to the patient’s advanced age and unstable renal function.

The patient was diagnosed with an odontogenic brain abscess due to *Fusobacterium necrophorum*, which was isolated from a culture of pus collected during the procedure. No bacteria were isolated in the aerobic culture. The antimicrobial susceptibility results for isolated *F. necrophorum* are presented in Table 2.

**Table 2:** Susceptibility pattern of antibiotics for Fusobacterium necrophorum. Susceptibility results were assessed based on clinical and laboratory institute testing standards M100 and S30 using the broth microdilution method.

Antimicrobial agents	Susceptibility
Cefazolin	Sensitive
Ceftriaxone	Sensitive
Cefepime	Sensitive
Meropenem	Sensitive
Levofloxacin	Sensitive
Clindamycin	Sensitive
Fosfomycin	Sensitive
Aztreonam	Sensitive

Following drainage, neurological symptoms observed on admission, such as aphasia and dysarthria, showed improvement, and anaphylaxis did not recur during MNZ therapy.

**Figure 1:**
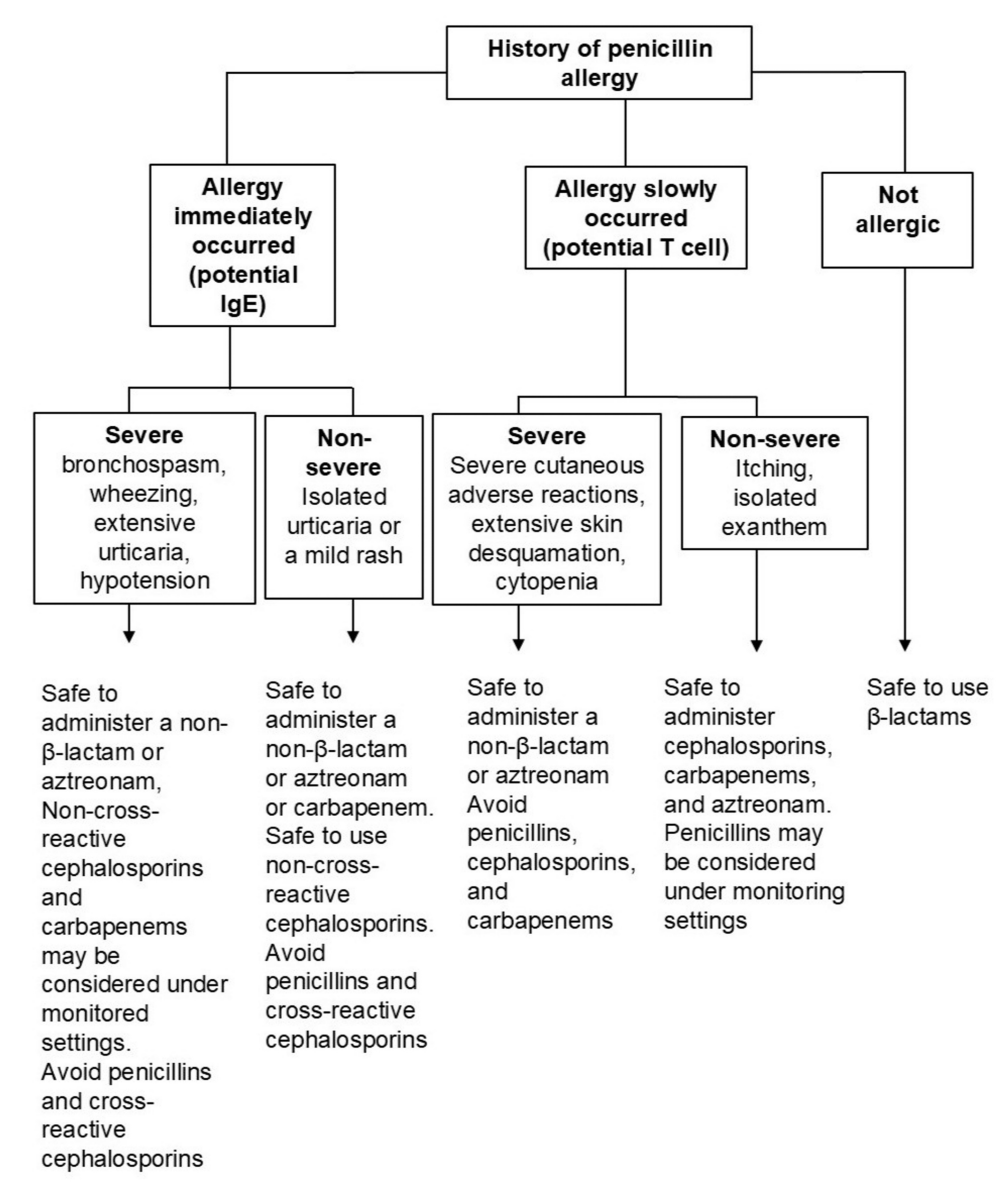
Treatment algorithm for patients with a history of penicillin allergy. Partially revised figure according to the Blumenthal et al. [7]  report.

We revised antibiotic therapy on day 14 because a residual abscess was detected on the head MRI. The antimicrobial spectrum was expanded because the involvement of aerobic bacteria, which MNZ does not cover, was suspected. According to Blumenthal et al. [7], non-β-lactam antibiotics or aztreonam (AZT) are recommended for patients with a history of severe PenA (see Figure 1).

**Figure 2:**
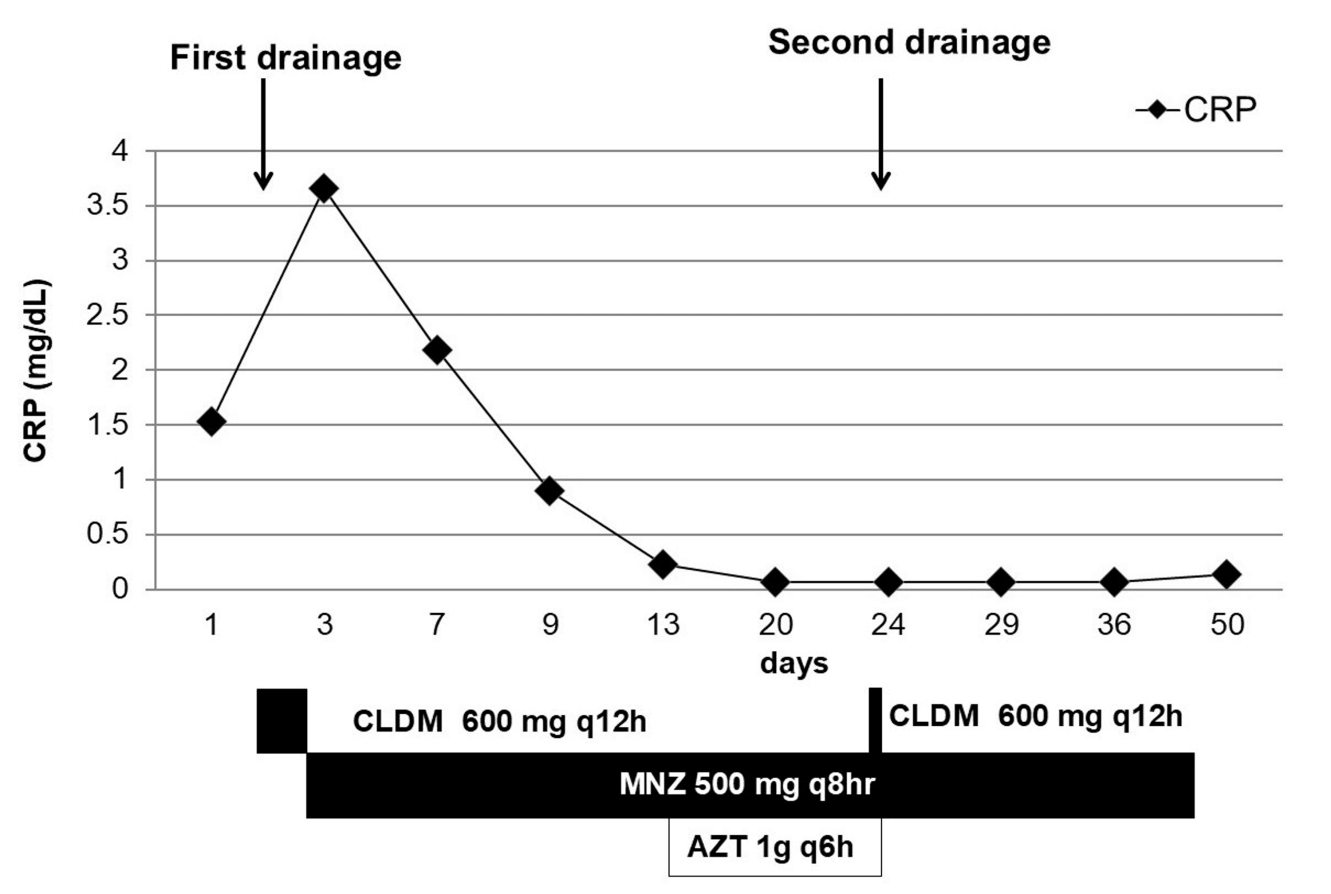
Timeline of the case and changes in an inflammatory marker level. CRP: C-reactive protein; CLDM: clindamycin; MNZ: metronidazole; AZT: aztreonam

In addition to its good penetration into the central nervous system, isolated *F. necrophorum* was susceptible to AZT. Therefore, combination therapy using AZT and MNZ was initiated on day 14 to expand the spectrum of antibiotics. The AZT dose was reduced to 1 g q6h due to impaired renal function. Figure 2 shows the antibiotics administered and changes in C-reactive protein (CRP) levels during hospitalization.

**Figure 3:**
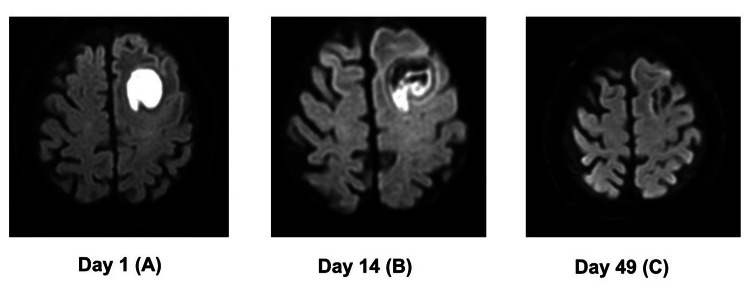
Changes in head MRI. (A) Preoperative head MRI showing an abscess in the left frontal lobe. (B) Postoperative image. The brain abscess decreased in size following drainage and antimicrobial therapy. However, a residual abscess remained. (C) Final MRI scan. The clearance of the abscess was confirmed following 45 days of MNZ therapy and further drainage. MRI: magnetic resonance imaging; MNZ: metronidazole

Although anaphylactic symptoms did not recur, AZT was discontinued on day 23 due to nausea. Drainage was performed again on the same day using the initial drainage wound. CLDM was intravenously administered in combination with MNZ to prevent SSI after the second procedure. MNZ was continued for 45 days. Head MRI on day 49 revealed that the brain abscess had completely cleared. Figure 3 shows changes in head MRI during hospitalization.

The patient was discharged on day 90 because there were no neurological aftereffects of the brain abscess.

## Discussion

Many patients have a history of PenA due to the high frequency of administration of penicillin. Therefore, a more detailed understanding of alternative antimicrobial agents is essential to provide safe and effective treatment for these patients.

Anaphylaxis is defined as an immediate hypersensitivity reaction in which the causative agent binds to IgE antibodies, resulting in the release of inflammatory cytokines [8]. Our patient had a history of severe PenA; he previously developed hypotension and loss of consciousness immediately after receiving penicillin antibiotics. Blumenthal et al. [7] reported an algorithm for alternative antimicrobial therapy among patients with PenA. The administration of non-β-lactam antibiotics or AZT is recommended for patients with a history of severe PenA. Cephalosporin with low cross-reactivity or a carbapenem antibiotic is also recommended, with close monitoring of the circulation. In the present case, the administration of cephalosporins was avoided because there are no intensive care physicians who manage anaphylaxis in our institution. Meropenem (MEPM) is an attractive agent for the treatment of brain abscesses in patients with a history of severe PenA because it has an extremely low rate of cross-sensitivity with penicillin antibiotics. However, it is impossible to administer a sufficient dosage of MEPM for six to eight weeks because the nationwide supply of MEPM in Japan was interrupted for approximately six months beginning in August 2022 due to manufacturing process deficiencies at the producing companies [9]. Therefore, the patient received MNZ monotherapy because the physician emphasized the avoidance of anaphylaxis.

*F. necrophorum*, an oral flora bacterium, is classified as an anaerobic, gram-negative, rod-shaped bacterium. It has been reported to cause lung abscesses and periodontal disease. Brain abscesses due to *Fusobacterium* species have a lower mortality rate than those of other bacteria due to the low incidence of loss of consciousness [10]. The causative bacteria of brain abscess in the present case were considered to be *F. necrophorum* only because the aerobic culture was negative, whereas *F. necrophorum* was isolated under anaerobic conditions. In addition to early drainage, the outcome of this case was favorable due to the administration of MNZ, which targeted oral anaerobic bacteria.

The mechanisms underlying the development of brain abscess include direct contamination through a neurosurgical procedure or head trauma and hematogenous spread due to bacteremia [1]. These typical entry routes did not apply to the present case because there was no history of neurological surgery or head trauma, and bacteria were not isolated from a blood culture. Dental or odontogenic infection has been reported as an underlying cause of brain abscess, but it occurs less frequently than other mechanisms. Ewald et al. [11] proposed the following criteria for diagnosing a brain abscess oriented from odontogenic infection: (i) the absence of an alternative source of bacteremia; (ii) the presence of oral microflora bacteria in the abscess; and (iii) clinical signs of active dental disease. The present case met these criteria. Moreover, odontogenic brain abscesses are well known to be frequently caused by anaerobic bacteria, including *Fusobacterium* species, as previously reported [12]. These observations support our interpretation that* F. necrophorum* may be the predominant or even sole pathogen in brain abscesses of oral origin. *F. necrophorum* may have entered the body during tooth extraction, which caused the brain abscess. The recommended empiric therapy for community-acquired odontogenic brain abscess is third-generation cephalosporin plus MNZ [6]. There are no established treatment regimens for cases in which a third-generation cephalosporin cannot be used because of allergy. Therefore, MNZ may be combined with a non-β-lactam antibiotic to cover aerobic bacteria based on the findings of a culture test.

There are several issues in the present case that need to be addressed. A previous study indicated that 47% of patients with odontogenic brain abscesses were infected with multiple bacteria, including streptococci [12]. As the physician’s primary concern in the present case was avoiding anaphylaxis, MNZ monotherapy was initiated as an empirical treatment. Therefore, there was a risk of treatment failure because empiric therapy did not cover aerobic bacteria (e.g., streptococci). Furthermore, a drug susceptibility test for MNZ was not performed on isolated *F. necrophorum *due to the lack of laboratory equipment to perform susceptibility tests for anaerobic bacteria in our institution. Previous studies reported that the susceptibility rate of *Fusobacterium* species to MNZ was 100% in Japan [13]. Therefore, it was presumed that the isolated *F. necrophorum* was susceptible to MNZ in this patient.

## Conclusions

We described an odontogenic brain abscess in a patient with a history of PenA. This case underscores the importance of obtaining a detailed allergy history and individualizing antibiotic therapy in elderly patients. Interdisciplinary collaboration between neurosurgeons and ID-specialized pharmacists enabled safe management without recurrent anaphylaxis. In this case, MNZ monotherapy was chosen because of the patient’s penicillin allergy and the results of microbiological cultures. However, this regimen is not the standard of care for brain abscess. Although this case report may help inform treatment strategies for brain abscess in patients with PenA, caution is warranted when extrapolating this approach to other patients. The accumulation of similar case reports may inform future guidelines for the management of brain abscesses in patients with severe PenA. The most important learning point of this case is that physicians must confirm histories of antibiotic allergies and adjust treatment plans based on the severity of symptoms, particularly for patients with a history of PenA.
